# Supplementary material for: PhotoFiTT: a quantitative framework for assessing phototoxicity in live-cell microscopy experiments
Source: Nat Commun. 2025 Dec 13;16:11401. doi: 10.1038/s41467-025-66209-6 (PMC12738901; doi:10.1038/s41467-025-66209-6)
Supplement: Supplementary file 1 — Supplementary Information [file 41467_2025_66209_MOESM1_ESM.pdf]

Note S1: Setting up and using PhotoFiTT in diverse systems

PhotoFiTT is designed to be a versatile and adaptable framework that can be integrated into a wide range of existing live-cell imaging workflows. Its primary aim is to efficiently evaluate and mitigate phototoxicity. This detailed guide walks researchers through the steps required for implementing PhotoFiTT, reflecting the components illustrated in Figure 2a.

A. Cell Culture and Synchronisation. The first step in the PhotoFiTT workflow involves preparing the cell samples. This process can be adapted to various cell types, but for optimal results, adherent cell lines capable of division should be used. The following steps can be overviewed in Figure 2a diagram blocks: "Unsynchronised cell culture", "Synchronised cell culture", "Cell synchronisation drug" and "Drug removal".

1. Cell seeding: Seed cells on an appropriate imaging substrate, such as an 8-well chambered cover glass. The seeding density should be optimised for your specific cell type, but as a starting point, consider using 6×10^4 cells per well if using CHO cells. Optimal cell density is crucial for obtaining high-quality images for analysis. Overly dense cell populations can complicate image analysis due to overcrowding and may inhibit cells from re-entering interphase after division due to limited space. Conversely, sparsely populated fields may yield insufficient data for robust statistical analysis.

2. Synchronisation (optional): Incubate cells with 10 µM RO-3306 (a CDK1 inhibitor) for 16−18 hours. This arrests cells at the G2/M boundary, allowing for a coordinated release into mitosis. It is not advisable to incubate cells for longer than 20 hours in RO-3306 as it can induce apoptosis[1].

3. Unsynchronised option: After seeding, allow cells to grow for the same duration as the synchronised cell population (16−18 hours). Recommendation: Conduct simultaneous experiments with both synchronised and unsynchronised cell populations. This approach allows for the fine-tuning of synchronisation timings and provides insights into the impact of synchronisation on phototoxicity sensitivity for the specific cell type under study.

B. Photodamage Irradiation. This phase replicates the illumination conditions encountered by cells in fluorescence microscopy experiments, corresponding to the "Phototoxicity Irradiation" segment depicted in Figure 2a. It's designed to closely mimic the irradiance and light doses typical in such experiments, providing a realistic assessment of potential phototoxic effects.

1. Microscope Configuration: Employ a fluorescence microscope outfitted with light sources that can replicate the specific illumination conditions required for assessing phototoxicity. This setup is crucial for accurately simulating the light exposure patterns cells will experience during the experiments.

2. Power Calibration Protocol: Initiate each experimental session by calibrating the photodamage irradiance to adjust the light dose. This step is paramount to guarantee uniformity in experimental conditions. Employ a power meter to accurately measure the irradiance (W/cm^2) at the sample plane, ensuring the microscope's internal chamber has stabilised at 37 °C. Control for the fact that irradiance can vary over time. This calibration is critical before commencing each experiment to maintain consistency and reliability in results.

3. Illumination Conditions: Configure several fields of view within a single well, assigning unique illumination conditions to each well. This strategy enables the simultaneous evaluation of various light exposure scenarios, facilitating a comprehensive analysis of phototoxic effects across different intensities and durations.

4. Light exposure Process:

- Area Selection: Ensure the selection of distinct, non-overlapping fields for each exposure condition to prevent double irradiation of the same area. Avoid tiling patterns that might lead to such overlaps.
- Location Tracking: Record the coordinates of each illuminated area. This information is crucial for subsequent time-lapse imaging, ensuring accurate follow-up on the exposed regions.
- Illumination Application: Implement the predefined illumination protocols precisely for each designated area, adhering to the established parameters for irradiance and light dose.

- Media Change for Synchronised Cells: For cells undergoing synchronisation, wash thoroughly with PBS twice before media replacement. We use a custom-designed 3D-printed multi-syringe adaptor (available on the PhotoFiTT GitHub repository) for efficient and uniform media changes across multiple wells simultaneously. This adaptor is compatible with an 8-well chamber, allowing for simultaneous media changes in 4 wells (Figure S6). The consistency of the media exchange process is important for accurate comparison of cell division timings across different experimental setups. Consider that the interval between synchronisation drug removal and the onset of mitotic cell rounding is typically under 10 minutes.

C. Live-cell Imaging. Following the completion of the light irradiation stage, the experimental protocol transitions to the timelapse imaging phase. This critical step, denoted as "Acquisition" in the schematic presented in Figure 2a, involves capturing sequential images over time to monitor and document the cellular responses to the previously applied light conditions.

1. Media Exchange Protocol: Transition the cells to an imaging-optimised medium to enhance both image quality and cell health during observation. Use phenol-red free Fluorobrite DMEM, fortified with 4 mM GlutaMAX for sustained cellular metabolism, 42 µM gentamicin to prevent microbial contamination, and 10% FBS to support cellular growth and viability.

2. Microscope Configuration: Use a high-quality objective that allows to resolve a large number of cells. Ensure the imaging chamber is regulated to maintain an environment that mirrors physiological conditions, with a constant temperature of 37°C and a 5% CO_2 concentration.

3. Acquisition Parameters: Use low-irradiance brightfield illumination to minimise phototoxicity. Schedule image acquisition over a span of 6–8 hours, with images captured every 4 minutes. This frequency balances the need for detailed temporal resolution while constraining phototoxicity. Select a magnification that allows for clear visualisation of individual cells and their mitotic stages (e.g. a 20X magnification objective).

4. Multi-Position Imaging Protocol: Configure the microscope for automated imaging of all areas subjected to light exposure, as well as designated control regions. For experiments incorporating a tiling strategy, align the regions of photodamage irradiation with those selected for detailed imaging.

Recommendation: Implement adaptive focus control, if available, to ensure stable focus over extended imaging sessions. Cellular focus can vary, especially between interphase cells and those in mitotic rounding or division. Identify cells at the mitotic rounding stage to fine-tune the focus, aiming for a balance that accommodates both cell states effectively. This adjustment should be completed prior to initiating photodamage irradiation and drug removal processes, to prevent any delays in live-cell imaging that could impact the integrity of the experimental results.

D. Image Analysis Workflow. The PhotoFiTT image analysis pipeline comprises several steps to extract quantitative data from time-lapse images. This workflow integrates deep learning techniques with traditional image processing methods to provide a comprehensive analysis of cellular behaviour under various photodamage irradiation conditions. These steps correspond to the workflow blocks "Data annotation", "Identification", "Virtual nuclei staining", "Cell nuclei segmentation", "Mitotic rounding detection", "Calculation of cell activity" illustrated in Figure 2a.

1. Cell Detection and Quantification (deep learning-based image analysis): We used virtual staining. Alternatively, one could use existing pipelines or pre-trained models for cell segmentation when available. This processing is only applied to the first time point of each video.

- Virtual Staining: Apply a Pix2Pix model or an alternative deep learning approach to generate virtual nuclear stains from brightfield images. This step is crucial for label-free cell detection and is performed only on the first frame of each video sequence. See Methods section B for the experimental acquisition of ground truth data and Methods section D for the model training. When reproducing our setup, use our pre-trained Pix2Pix model.
- Nuclei Segmentation: Use the pretrained StarDist-versatile model provided by the original authors to segment individual nuclei in the virtually stained images, providing accurate cell counts and positions.

- Initial Cell Quantification: The number of detected nuclei (≈ 125 cells per FOV estimated in average from experimental counts) serves as the baseline cell count for each field of view, enabling tracking of population dynamics over time.

2. Mitotic Cell Identification (deep learning-based image analysis):

- CHO-specific Detection: For Chinese Hamster Ovary (CHO) cells, employ the specialised StarDist-CHO model to identify cells in the mitotic rounding phase, leveraging its training on the unique morphology of dividing CHO cells.
- For other cell lines: For other cell types or experimental conditions, manually annotate a representative image set and train a new StarDist model following the protocol outlined in the Methods.

3. Cell Size Analysis and Classification:

- Morphometric Measurements: Exploit the instance segmentations from StarDist-CHO to calculate cell areas and estimate cell diameters.
- Cell Stage Classification: Categorise cells as "mother" (diameter > 18 µm) or "daughter" (diameter ≤ 18 µm) based on their size, enabling tracking of cell division progression.

4. Quantification of Cellular Activity:

- Image Preprocessing: Enhance brightfield images through illumination normalisation, noise reduction, and contrast enhancement to optimise subsequent analysis.
- Dynamic Activity Measurement: Compute frame-to-frame differences to quantify overall cellular activity, capturing subtle changes in cell morphology and position.
- Cumulative Activity Assessment: Calculate the total cellular activity over the entire imaging period to assess long-term effects of light exposure.

E. Implementation Guidelines

1. Deep Learning-based Image Processing:

- Local Processing: Install DL4MicEverywhere[2] and execute the appropriate notebooks for Pix2Pix and StarDist models.
- Cloud-based Processing: Use ZeroCostDL4Mic[3] notebooks on Google Colab for Pix2Pix and StarDist implementations.
- Pre-trained Models: Access our validated models from Zenodo for immediate use or as starting points for transfer learning.

2. Data Analysis and Processing:

- Environment Setup: Configure the required Python environment as specified in the PhotoFiTT repository.
- Data Organisation: Structure raw videos and generated masks according to the provided template.
- Analysis Execution: Employ the provided Jupyter Notebooks to process data and replicate the analysis pipeline.

3. Quality Control and Validation:

- Manual Verification: Manually check a subset of images to ensure accurate cell detection and classification.
- Reproducibility Assessment: Compare results across experimental replicates to ensure consistency and reliability.

F. Data Interpretation and Optimisation. The final phase of the PhotoFiTT workflow involves interpreting the results and using them to refine your imaging protocols. The following analysis is supported by the output of PhotoFiTT's Jupyter notebooks. It represents the "Data analysis" workflow illustrated in Figure 2a.

1. Mitotic Timing Analysis: Plot the distribution of mitotic rounding events over time for each condition. Identify the peak division time for control populations (typically around 50 minutes post-synchronisation release). Quantitatively assess delays in this peak for different light exposures and wavelengths.

2. Cell Size Dynamics Evaluation: Monitor the temporal evolution of mother and daughter cell proportions. Identify delays in daughter cell emergence, indicative of division slowdown. Identify conditions leading to persistent large cell populations, suggesting potential cell cycle arrest.

3. Cellular Activity Assessment: Perform a comparative analysis of cumulative activity levels across different experimental conditions. Interpret reduced activity as a potential indicator of cellular stress or compromised viability.

4. Phototoxicity Threshold Determination: Identify the minimum light dose that induces detectable alterations in mitotic timing, cell size distribution, or cellular activity. Use these thresholds as illumination constraints for designing imaging protocols.

Note S2: Optimising Live-Cell Imaging Experiments with PhotoFiTT

To maximise the efficacy of PhotoFiTT in live-cell microscopy protocol design, one should implement a systematic approach beginning with comprehensive baseline measurements. These initial assessments should encompass a spectrum of illumination conditions directly relevant to typical experimental parameters, including varying wavelengths, irradiances, and light doses. This baseline characterisation provides quantitative metrics indicating where current protocols fall on the phototoxicity-resolution continuum, establishing a reference point for subsequent optimisation.

Following baseline establishment, iterative protocol refinement becomes essential. The quantitative nature of PhotoFiTT's measurements enables evidence-based optimisation rather than heuristic approaches. Multiple optimisation cycles may be necessary, systematically adjusting parameters such as irradiance, light dose, acquisition frequency, and wavelength selection. This iterative process should continue until an optimal balance between image quality and cellular physiological integrity is achieved, as indicated by minimal alterations in mitotic timing, cell morphology and overall cellular activity.

PhotoFiTT assessment should be integrated into routine experimental workflows as a continuous monitoring strategy. This integration becomes particularly crucial when introducing experimental variables that may alter phototoxicity sensitivity, including novel cell lines, modified culture conditions, experimental compounds with potential photosensitising effects, new fluorophores or labelling strategies, and implementation of different imaging modalities or hardware upgrades. Regular phototoxicity assessment enables timely protocol adjustments before compromised data quality occurs.

Finally, comprehensive reporting of PhotoFiTT-derived phototoxicity assessments in methods sections and supplementary materials should become standard practice. By including quantitative metrics of illumination conditions, cellular responses, and optimisation strategies, researchers enhance experimental reproducibility and enable more meaningful cross-laboratory comparisons. This approach transforms phototoxicity from a qualitative concern to a rigorously defined experimental parameter, elevating the overall quality and reliability of live-cell microscopy studies throughout the research community.

Note S3: Practical implementation examples

Core Phototoxicity Equation

The fundamental relationship governing phototoxicity effects is defined as:

$$Total\ Dose\ \left(\frac{J}{cm^2}\right) = Irradiance\ \left(\frac{W}{cm^2}\right) \times\ Exposure\ Time\ (s)$$

This enables direct comparison of illumination strategies through proportional power-time compensation.

Example 1: Widefield Imaging Optimisation

Time-lapse imaging of CHO cells labelled with MitoTracker green

Baseline Parameters (High Irradiance):

Irradiance = 5 W/cm^2

Safe dose limit = 5 J/cm^2 (estimated from PhotoFiTT benchmark)

Max safe light dose:

$$\frac{5\ J/cm^2}{5\ W/cm^2} = 1\ s$$

Using 10ms light exposure, the maximum number of illumination time-points preserving safe conditions is estimated as:

$$\frac{1\ s}{0.01\ s/exposure} = 100\ frames$$

Optimised Parameters (Low Irradiance + Image denoising):

Irradiance = 0.5 W/cm^2 (10X reduction)

New safe light dose:

$$\frac{5\ J/cm^2}{0.5\ W/cm^2} = 10\ s$$

With denoising: 12 s total

New achievable light dose using 10ms light exposure:

$$\frac{10\ s}{0.01\ s/exposure} = 1000\ frames$$

Example 2: Cell Line Specific considerations

CHO cells

Critical dose thresholds:

Wavelength	Threshold Dose	Max Safe Duration
385 nm	0.6 J/cm^2	12 minutes
475 nm	6 J/cm^2	120 minutes
630 nm	60 J/cm^2	12 hours

HeLa Cells

2x higher tolerance for 385 nm light dose

Recommended safety buffer:

$$Adjusted\ Dose = 2\ \times\ CHO\ Threshold$$

Example 3: Multi-Channel Imaging Protocol

The maximum safe light dose for a multichannel imaging experiment is estimated as

$$Multichannel\ Threshold\ \left(\frac{J}{cm^2}\right) = \sum_{i=1}^{n} w_i * Threshold_i\ \ where\ \sum_{i=1}^{n} w_i \leq 1$$

where i represents each wavelength channel, n is the total number of channels to illuminate using fluorescence, and w_i is the usage of the maximum safe light dose from channel i that contributes to the overall dose and is calculated as:

$$w_i = \frac{Dose_i}{Threshold_i}$$

Example Implementation for simultaneous GFP/RFP imaging:

**Channel**	**λ (nm)**	**Dose (J/cm^2)**	**Threshold (J/cm^2)**	**Usage ($100*w_i$)(%)**
**GFP**	488	3.0	6.0	50%
RFP	561	4.5	9.0	50%
**Total**				100%

Example 4: Temporal Optimisation Guidelines

Time-lapse imaging: Estimate the maximum exposure time for a given irradiance

$$Exposure\ Time = \frac{Total\ Safe\ Exposure\ Time}{Desired\ Frames}$$

3D imaging: Allocate dose proportionally

$$Per - slice\ Dose = \frac{Total\ Dose}{Number\ of\ Z\ slices}$$

Supplementary Bibliography

1. Vassilev, L. T. Cell Cycle Synchronization at the G $_2$ /M Phase Border by Reversible Inhibition of CDK1. *Cell Cycle* **5**, 2555–2556 (2006).
2. Hidalgo-Cenalmor, I. *et al.* DL4MicEverywhere: deep learning for microscopy made flexible, shareable and reproducible. *Nature Methods* 1–3 (2024).
3. von Chamier, L. *et al.* Democratising deep learning for microscopy with ZeroCostDL4Mic. *Nat Commun* **12**, 2276 (2021).
4. Wildanger, D., Rittweger, E., Kastrup, L. & Hell, S. W. STED microscopy with a supercontinuum laser source. *Opt. Express* **16**, 9614 (2008).
5. Diekmann, R. *et al.* Optimizing imaging speed and excitation intensity for single-molecule localization microscopy. *Nat Methods* **17**, 909–912 (2020).

6. Grotjohann, T. *et al.* Diffraction-unlimited all-optical imaging and writing with a photochromic GFP. *Nature* **478**, 204–208 (2011).
7. Icha, J., Weber, M., Waters, J. C. & Norden, C. Phototoxicity in live fluorescence microscopy, and how to avoid it. *Bioessays* **39**, (2017).
8. Culley, S., Tosheva, K. L., Matos Pereira, P. & Henriques, R. SRRF: Universal live-cell super-resolution microscopy. *The International Journal of Biochemistry & Cell Biology* **101**, 74–79 (2018).
9. Kwakwa, K. *et al.* easySTORM: a robust, lower-cost approach to localisation and TIRF microscopy. *Journal of Biophotonics* **9**, 948–957 (2016).
10. Li, D. *et al.* Extended-resolution structured illumination imaging of endocytic and cytoskeletal dynamics. *Science* **349**, aab3500 (2015).
11. Schermelleh, L. *et al.* Super-resolution microscopy demystified. *Nat Cell Biol* **21**, 72–84 (2019).

Supplementary figures

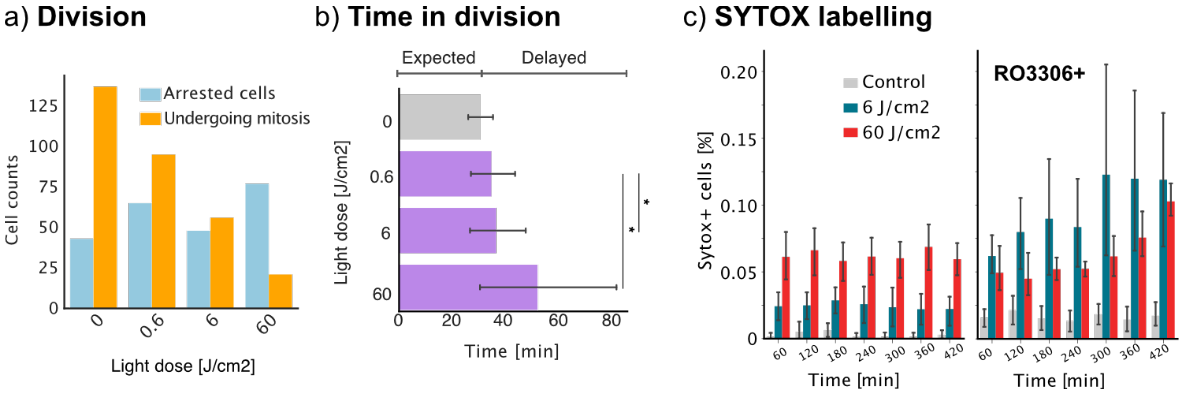

Fig. S1. Impact of phototoxicity on non-synchronised cell populations. Figure presents the effects of phototoxicity on non-synchronised adherent mammalian (Chinese Hamster Ovary - CHO) cell populations under near-UV (385 nm) light dose. a) Relationship between light dose and cell fate, showing a decrease in cell division and an increase in cell arrest with higher UV doses. b) Dose-dependent delay in cell division is quantified by measuring the time from mitotic rounding to the emergence of two distinct daughter cells, revealing longer delays at higher near-UV doses. c) Comparison of apoptotic cell rates (indicated by SYTOX-positive staining) between synchronised and non-synchronised cell populations under near-UV radiant exposure highlights higher vulnerability of synchronised populations to phototoxic damage. In 385nm (0 (n=5), 0.6 (n=5), 6 (n=5), and 60 (n=5)). A total of 1 biological replicate with 5 technical replicates was done. In b-c) data are represented as mean values +/- 95% Confidence Interval. Statistical significance estimated by Kolmogorov-Smirnov hypothesis test with * (p-value<0.05), ** (p-value<0.01), *** (p-value<0.001), **** (p-value<0.0001).

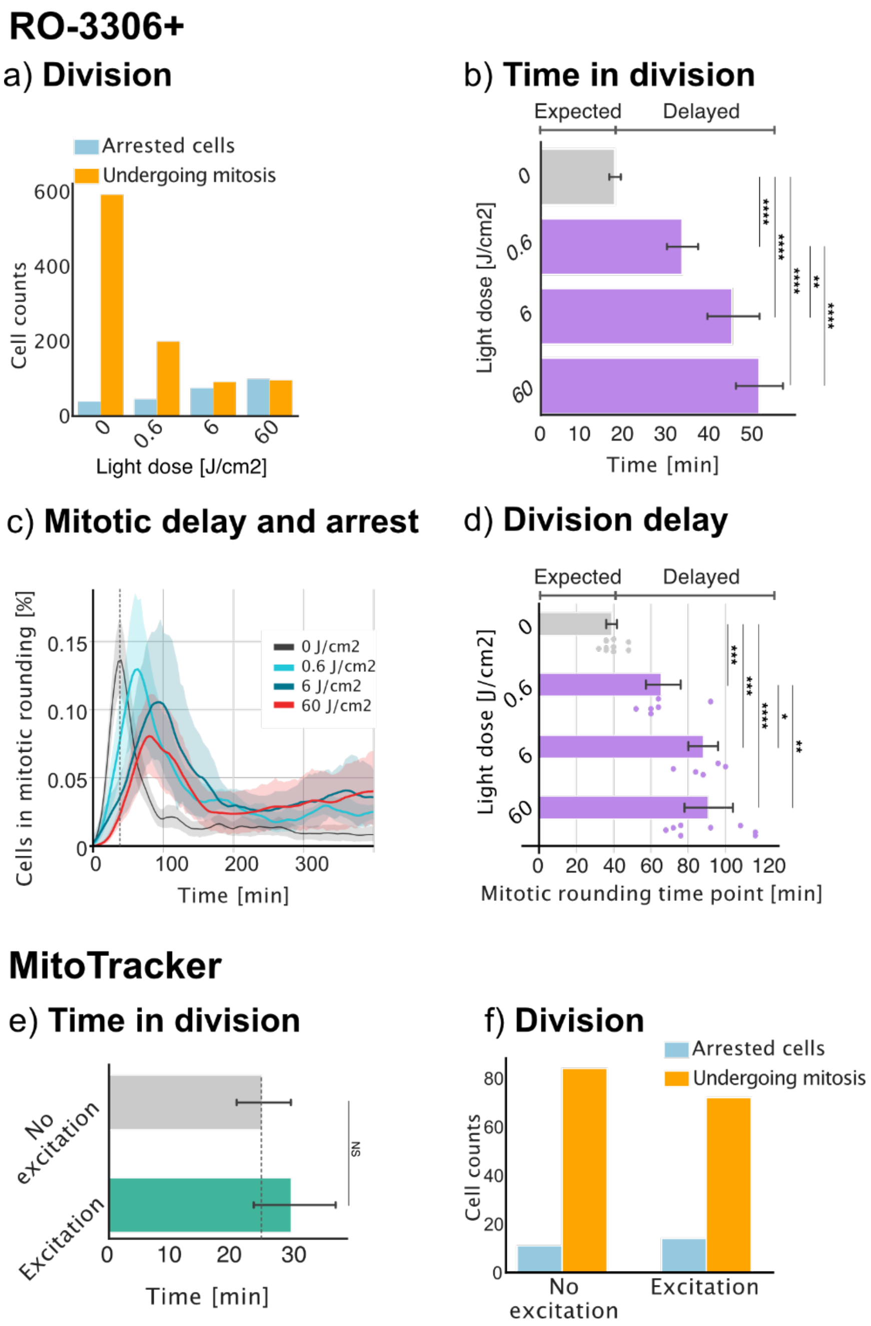

Fig. S2. Results for manually annotated videos. The figure presents the effects of phototoxicity on manually annotated time lapse videos of adherent mammalian (Chinese Hamster Ovary - CHO) cell populations. a-d) Show the delay in mitosis and mitotic ratios of CHO cells under near-UV (385 nm) light exposure. a) The relationship between light dose and cell fate shows a decrease in cell division and an increase in cell arrest with higher UV doses. b) Dose-dependent delay in cell division is quantified by measuring the time from mitotic rounding to the emergence of two distinct daughter cells, revealing longer delays at higher near-UV doses. c) Temporal distribution of mitotic cell rounding in synchronised populations exposed to varying doses of 385 nm (near-UV) light (0.6, 6, and 60 J/cm2). d) Quantification of mitotic rounding delays. The control population peaks at t =40 minutes. e-f) Shows the mitotic delay caused by phototoxicity on CHO cells labelled with MitoTracker Red and Green, illuminated with 475 nm. e) Fluorophore dependent delay in cell division is quantified by measuring the time from mitotic rounding to the emergence of two distinct daughter cells, revealing longer delays when the fluorophore is excited. f) The relationship between excited fluorophores and cell fate shows a decrease in cell division and an increase in cell arrest with excited fluorophores. In 385nm (0 (n=17, 5 replicas), 0.6 (n=6, 2 replicas), 6 (n=22, 3 replicas), and 60 (n=9, 3 replicas)). A total of 5 biological replicates were done. For MitoTracker, 475nm excitation (0 (n=10, 2 replicas), Excitation (n=10, 2 replicas). A total of 2 biological replicates were done. In b) and d-e) data are represented as mean values +/- 95% Confidence Interval. Statistical significance estimated by Kolmogorov-Smirnov hypothesis test with NS: no significance (p-value>0.05), * (p-value<0.05), ** (p-value<0.01), *** (p-value<0.001), **** (p-value<0.0001).

Vale!

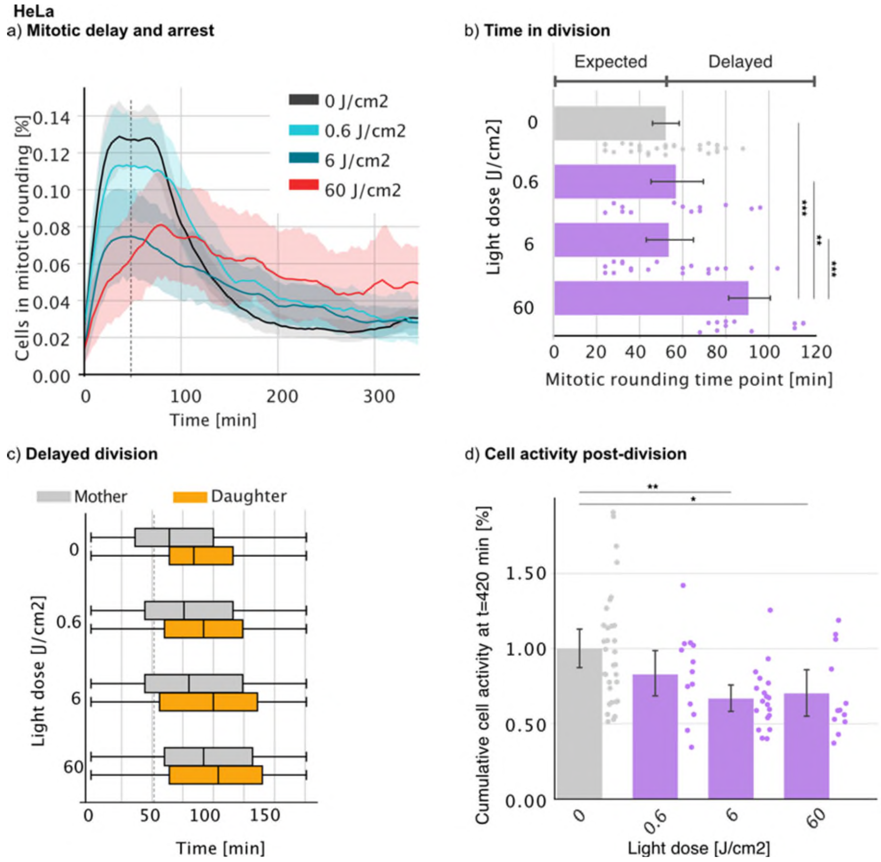

Fig. S3. PhotoFiTT results from HeLa cells. The PhotoFiTT workflow was tested on HeLa cells following the same mitosis synchronisation conditions depicted in (Figure 2). a) Temporal distribution of mitotic cell rounding in synchronised populations exposed to varying doses of 385 nm (near-UV) light (0.6, 6, and 60 J/cm2). While similar to CHO cells, HeLa appear slightly more resistant to low doses of near-UV illumination (60 J/cm2). Exposed populations exhibit a dose-dependent delay in mitotic rounding, manifested as rightward shifts in the distribution peaks. b) Quantification of mitotic rounding delays across different doses. The control population peaks at t=50 minutes, representing the average mitotic rounding time. Exposure to 385 nm light induces a dose-dependent delay, that becomes apparent at high doses (60 J/cm2). c) Temporal analysis of mother and daughter cell populations across different irradiation doses. All light doses induce small delays in daughter cell appearance, with high doses causing the most pronounced effects when compared to the control. d) Cumulative cell activity over 7h hours post-exposure normalised for each replica. Showing differences with CHO cells, not all light exposures reduce overall cell activity, near-UV light causes a reduction in activity after 6 J/cm2. In 385nm (0 (n=32), 0.6 (n=13), 6 (n=19), and 60 (n=12)). A total of 2 biological replicates with 10 technical replicates were done. In b) and d) data are represented as mean values +/- 95% Confidence Interval. The boxes in c) show the quartiles of the dataset while the whiskers extend to the entire distribution, except for distributions where outliers surpass the inter-quartile range. Statistical significance estimated by Kolmogorov-Smirnov hypothesis test with * (p-value<0.05), ** (p-value<0.01), *** (p-value<0.001), **** (p-value<0.0001).

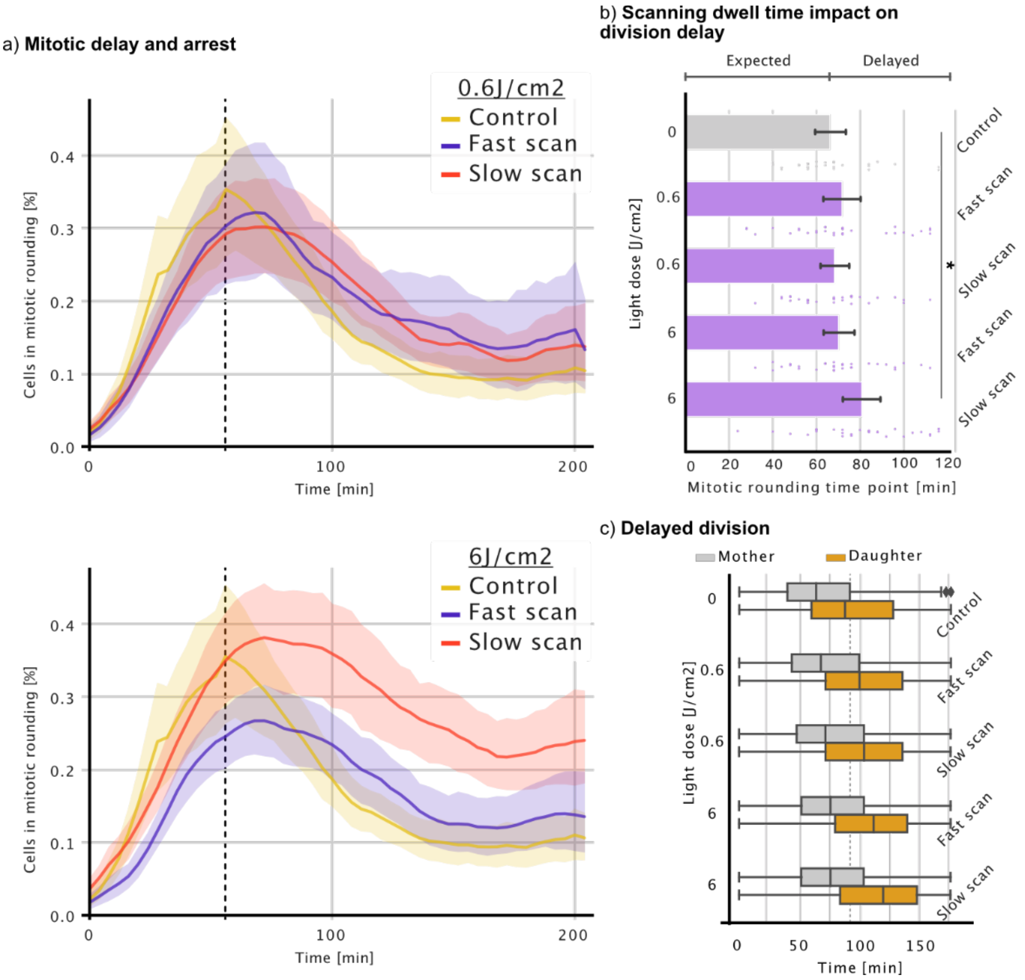

Fig. S4. Point-scanning confocal irradiation effect on cell cycle behaviour. The PhotoFiTT workflow was tested with point scanning confocal irradiation using a fast and a slow scanning dwell time. a) Temporal distribution of mitotic cell rounding in synchronised populations exposed to varying doses of 405 nm (near-UV) light (0.6 and 6 J/cm2). While the illumination patterns differ between widefield and confocal microscopy, efforts to maintain the same light dose were done. Exposed populations exhibit a dose-dependent delay in mitotic rounding, manifested as rightward shifts in the distribution peaks. Using a 0.6 J/cm2 light dose produced a modest effect, particularly using a fast dwell time. A higher dose of 6 J/cm2 resulted in marked delays during mitotic processes, particularly on slow dwell times, following the same trend as with lower doses. b) Quantification of mitotic rounding delays across different doses and dwell times. The control population peaks at t 60 minutes, representing the average mitotic rounding time. Exposure to 405 nm light induces a dose-dependent delay, that becomes apparent at high doses (6 J/cm2) and slow dwell times. c) Temporal analysis of mother and daughter cell populations across different irradiation doses and dwell times. All light doses induce small delays in daughter cell appearance, with high doses and slow dwell times causing the most pronounced effects when compared to the control. . In 385nm (0 (n=29), high power 0.6 (n=30), low power 0.6 (n=29), high power 6 (n=30) and low power 6 (n=30)). A total of 3 biological replicates with 10 technical replicates were done In b) and c) data are represented as mean values +/- 95% Confidence Interval. The boxes in c) show the quartiles of the dataset while the whiskers extend to the entire distribution, except for distributions where outliers surpass the inter-quartile range. Statistical significance estimated by Kolmogorov-Smirnov hypothesis test with * (p-value<0.05), ** (p-value<0.01), *** (p-value<0.001), **** (p-value<0.0001).

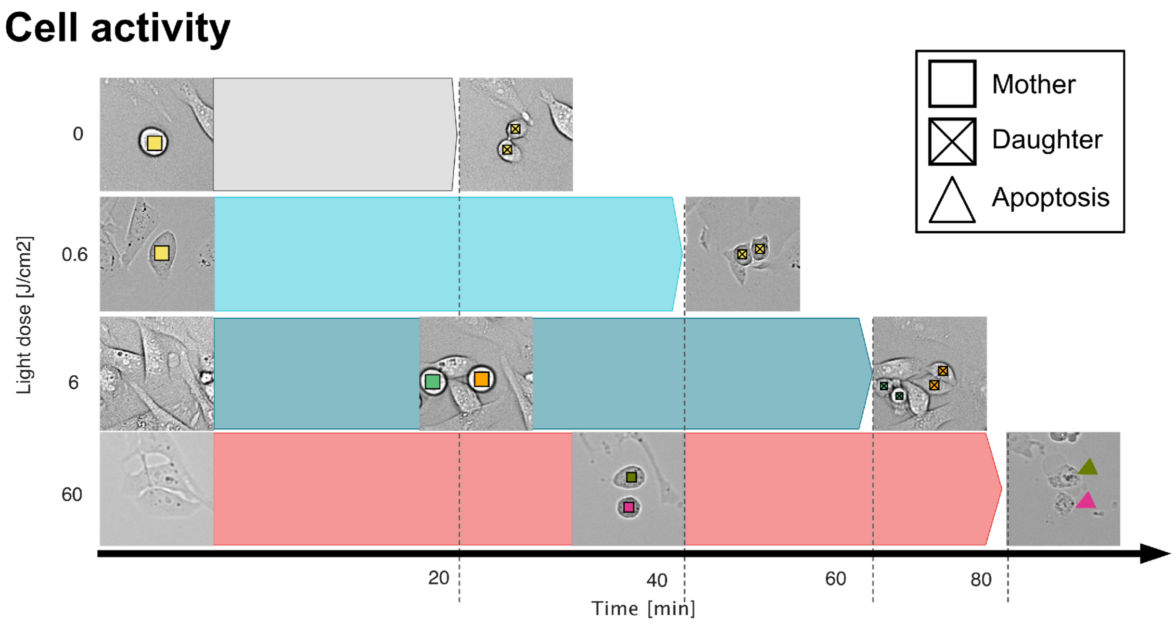

Fig. S5. Cell mitosis after light exposure. Temporal illustration of near-UV (385 nm) light dose effects on cell division timing, arrest, and apoptosis. In the absence or under low light exposure, mother cells (square) proceed to divide resulting in the formation of daughter cells (square with a cross inside). Following high light exposure, cells present delays in mitotic events resulting in delays or even apoptosis (triangle). Scale bar 50 µm.

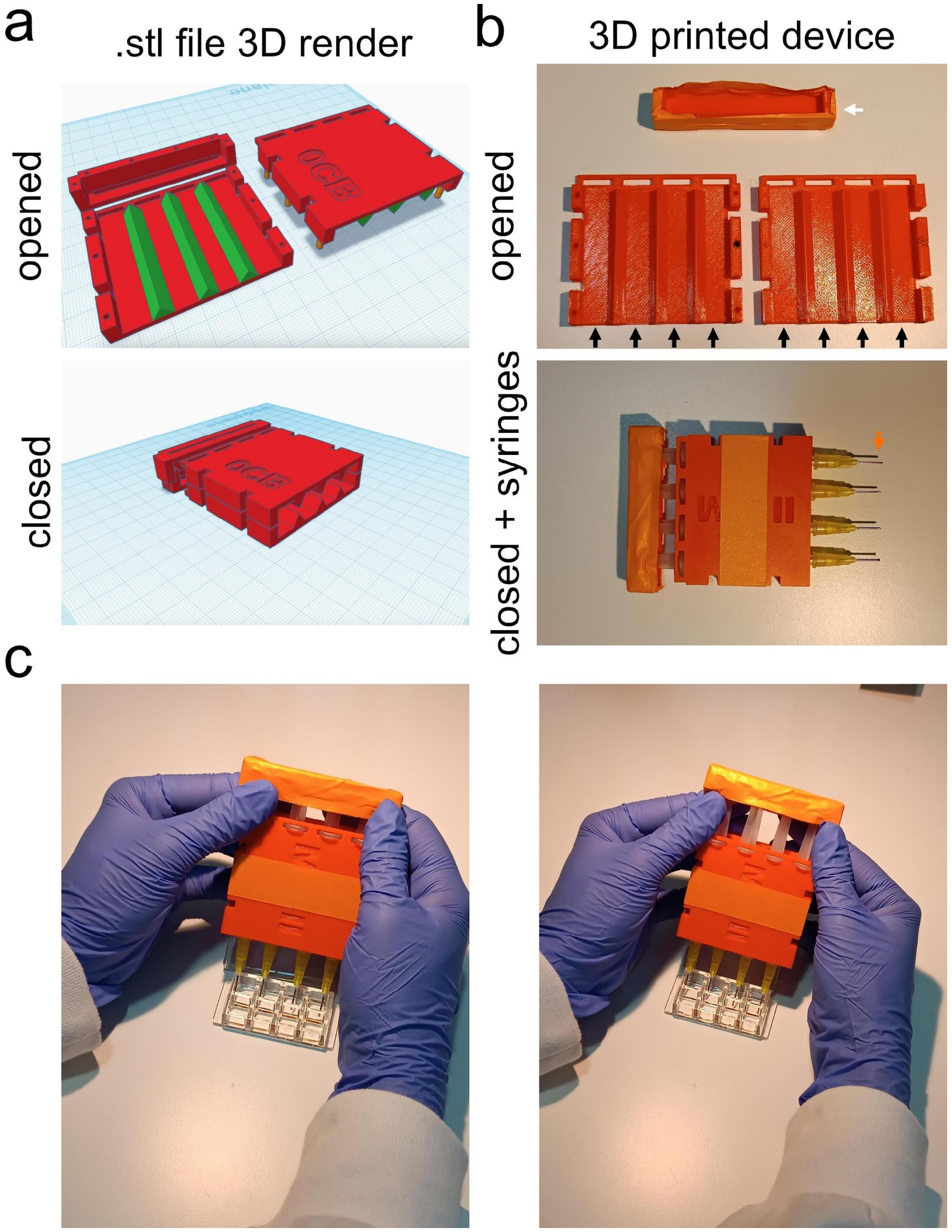

Fig. S6. 3D-printed multi-well syringe adaptor for efficient media changes. This custom device accommodates four 2.5 mL syringes for use with standard 8-well glass-bottom imaging chambers. a) 3D render of the .stl file in open and closed configurations. b) 3D-printed device with and without syringes. Black arrows indicate well spacing matching the 8-well chamber; white arrow shows the plunger thumb rest for uniform syringe motion; orange arrow points to recommended blunt needles for optimal media uptake. c) Demonstration of the adaptor's use on an 8-well chamber. The device was designed using TinkerCAD and printed with a Prusa MK4 3D printer. Additional securing with tape is optional.

Irradiation range (W/cm²)	Irradiation average (W/cm²)	Dose for 60s exposure average (J/cm²)	Microscopy modality	References
1000 - 20000	10000	600000	STED	Wildanger et al., 2008[36]
1000 - 10000	5000	300000	SMLM/RESOLFT	Diekmann et al., 2020[37]; Grotjohann et al., 2011[38]
100 - 5000	1000	60000	Confocal	Icha et al., 2017[2]
50 - 1000	100	6000	SRRF	Culley et al., 2018[39]
5 - 100	10	600	TIRF; SIM	Kwakwa et al., 2016[40]; Li et al., 2015[41]
0.5 - 100	5	300	LLS; Wide-field	Icha et al., 2017[2]; Schermelleh et al., 2019[42]

Table S1. Irradiation ranges and microscopy modalities for different fluorescence microscopy techniques. This table summarises the typical irradiation ranges, average irradiation intensities and average dose for 60 seconds of radiant exposure (dose (J/cm^2) = Irradiance (W/cm^2) x Time (s)) for various fluorescence microscopy techniques, along with corresponding references. STED: Stimulated Emission Depletion; SMLM: Single Molecule Localization Microscopy; RESOLFT: Reversible Saturable Optical Fluorescence Transitions; SRRF: Super-Resolution Radial Fluctuations; TIRF: Total Internal Reflection Fluorescence; SIM: Structured Illumination Microscopy; LLS: Lattice Light-Sheet.
