# Supplementary material for: PhotoFiTT: a quantitative framework for assessing phototoxicity in live-cell microscopy experiments
Source: Nat Commun. 2025 Dec 13;16:11401. doi: 10.1038/s41467-025-66209-6 (PMC12738901; doi:10.1038/s41467-025-66209-6)
Supplement: Supplementary file 2 — Description of Additional Supplementary Files [file 41467_2025_66209_MOESM2_ESM.pdf]

Description of Additional Supplementary Files

File Name: Supplementary Movie 1
Description: Detailed procedure for the PhotoFiTT experiment setup and image acquisition. This instructional video outlines the PhotoFiTT experimental setup and data acquisition process. Initially, standard adherent cells are seeded in suitable culture dishes. These cells are then synchronised using the CDK1 inhibitor, RO-3306, for a period of 16 to 18 hours to ensure uniform cell cycle progression. Prior to imaging, the microscope's excitation irradiance is calibrated to maintain consistency across all experiments. The cells are exposed to a predetermined dose of excitation light, simulating conditions encountered during fluorescence microscopy. Following light exposure, the cells are washed with PBS to remove any residual inhibitor, preparing them for live imaging. The video concludes with the initiation of live-cell acquisition, capturing the dynamic responses of cells to phototoxic stress.

File Name: Supplementary Movie 2
Description: Demonstrating the PhotoFiTT analytical workflow. This video provides a comprehensive walkthrough of the PhotoFiTT analytical process, utilising the Jupyter notebooks available in the PhotoFiTT GitHub repository (https://github.com/HenriquesLab/PhotoFiTT). The demonstration covers each step in detail, showcasing how to effectively use the provided tools for analysing phototoxicity effects on cells.

File Name: Supplementary Movie 3
Description: Dynamics of adherent cells after light exposure. Synchronised CHO cells following 385 nm (near-UV) light exposure. Non-exposed cells present a clearly defined peak in mitotic rounding and cell division (white arrow). Cells exposed to a dose of 0.6J/cm2 present a slight delay but can complete division (orange arrow). Cells exposed to a dose of 6J/cm2 present a delay in mitotic rounding. In most cases, the cells present aberrant division (blue arrow) or are unable to complete division (teal arrow). Cells exposed to the high dose of 60J/cm2 become arrested and can result in cell death (purple arrows).

File Name: Supplementary Movie 4
Description: Dynamics of HeLa cells after light exposure. Synchronised HeLa cells following 385 nm (near-UV) light exposure. Non-exposed, and cells exposed to a dose of 0.6J/cm2 present a similar behaviour without delays. Cells exposed to a dose of 6J/cm2 and 60J/cm2 become delayed and arrested, resulting in cell death in some cases.

File Name: Supplementary Movie 5
Description: Comparison of fast and slow point-scanning confocal speeds. Synchronised CHO cells following 405 nm light exposure. Non-exposed cells present a clearly defined trend in mitotic rounding and cell division. Cells exposed to a dose of 0.6J/cm2 present similar behaviours as non-exposed cells. Cells exposed to a dose of 6J/cm2 present a modest delay in mitotic rounding in slow scan speeds.

File Name: Supplementary Movie 6
Description: Effects of exciting MitoTracker Green FM during live cell acquisitions. Synchronised CHO cells following 385 nm (near-UV) light exposure. Non-exposed cells present physiological behaviour. Cells incubated with MitoTracker Green FM presents a very modest change in overall behaviour when compared to the Unlabelled and MitoTracker Red CMXRos controls.
